# Advances in paediatrics in 2019: current practices and challenges in allergy, endocrinology, gastroenterology, public health, neonatology, nutrition, nephrology, neurology, respiratory diseases and rheumatic diseases

**DOI:** 10.1186/s13052-020-00853-0

**Published:** 2020-06-29

**Authors:** Carlo Caffarelli, Francesca Santamaria, Virginia Mirra, Ermanno Bacchini, Angelica Santoro, Sergio Bernasconi, Giovanni Corsello

**Affiliations:** 1 Clinica Pediatrica, Department of Medicine and Surgery, Azienda Ospedaliera-Universitaria, University of Parma, Parma, Italy; 2 Department of Translational Medical Sciences, Federico II University, Naples, Italy; 3 Unità Polispecialistica Pediatrica Centro Medi Saluser, Parma, Italy; 4 Microbiome Research Hub, University of Parma, Parma, Italy; 5 Department of Sciences for Health Promotion and Mother and Child Care “G. D’Alessandro”, University of Palermo, Palermo, Italy

**Keywords:** Allergy, Endocrinology, Gastroenterology, Neonatology, Nutrition, Nephrology, Neurology, Public health, Respiratory diseases, Rheumatology

## Abstract

We highlight the main developments that have been published during the first semester of the last year in the Italian Journal of Pediatrics. We have carefully chosen information from numerous exciting progresses issued in the Journal in the field of allergy, endocrinology, gastroenterology, neonatology, nutrition, nephrology, neurology, public health, respiratory diseases and rheumatic diseases. The impact on the care of patients has been placed in the broader context of studies that appeared in other journals. We think that many observations can be used directly to upgrade management of patients.

## Background

This paper summarizes main advances that were reported in the field of allergy, endocrinology, gastroenterology, neonatology, nutrition, nephrology, neurology, public health, respiratory diseases and rheumatology over the first semester of 2019 in the Italian Journal of Pediatrics. The most accessed papers have been carefully selected and put in the context of studies that appeared in other journals.

## Review

### Allergy. 1-food allergy; 2-severe asthma; 3-vernal Keratoconjunctivitis

There is a continuous effort to improve the management of allergic diseases. Pathophysiology of food allergy is IgE-mediated, non IgE-mediated (cell-mediated), or mixed (IgE and cell-mediated). Manifestations of IgE-mediated reactions include rhinoconjunctivitis, asthma, rash, angioedema, urticaria, nausea, vomiting, abdominal pain, diarrhea, anaphylaxis. Trigger food is identified by medical history, IgE tests (skin prick tests and/or serum specific IgE antibodies) and oral food challenge that is the gold standard [1]. Avoidance of culprit food is the cornerstone of food allergy treatment. Oral immunotherapy (OIT) to cow’s milk, hen’s egg or peanut allergy is promising. However, it has been shown that OIT is not able to induce total or partial food tolerance in about 30% of children. Furthermore, the frequency of adverse events is high and duration of effectiveness unclear. Crisafulli G et al. [2] investigated the efficacy of add-on therapy with omalizumab a monoclonal anti-IgE antibody during OIT in 5 children with cow’s milk allergy. A combination treatment (OIT plus omalizumab) preceded by a pretreatment with omalizumab in 3 cases, was beneficial in most children and tolerability was good. Omalizumab is that has been approved for the treatment of severe persistent allergic uncontrolled asthma and spontaneous chronic urticaria. It has also been shown to be successful in chronic rhinosinusitis [2, 3]. Among allergies with a mixed (IgE and cell-mediated) pathophysiology, omalizumab could be helpful in improving atopic dermatitis [4] that shares with asthma, inflammatory mediators [5] and the response to allergen immunotherapy [6] but it did not improve eosinophilic esophagitis [2].

In asthmatic children, the prevention of recurrent symptoms is based on long-term therapy and avoidance of triggering factors including aeroallergens, food allergens [7], physical exercise, passive smoking, pollutants. Asthma control has been shown to be enhanced by communication interventions. However, almost all the asthmatic children are not enough engaged in discussions on management [8]. The SOUND project published a consensus for improving the communication to children and adolescents with severe asthma and their parents [9]. Recommendations are given to physicians on how welcome they have made children and parents. The knowledge of the context would be facilitated by asking questions on goal of the visit, daily life (diet, physical activity, relation with family members and school advancement), by understanding the view of the child, and by using child’s drawings to recognize his thoughts. Advices are given on management of emotions, and on how keeping relationships between the visits. It is recommended to avoid prohibitions since they can cause suffering and undesirably disturb the relationship.

Vernal keratoconjunctivitis (VKC) is an underestimate severe seasonal chronic inflammation that can lead to persistent damages [10]. A systematic review [11] has addressed the definition of diagnostic criteria and scoring system. A lack of standards for diagnosis was found. This mainly hampers the differentiation from seasonal allergic conjunctivitis that is often due to grass pollens [12]. This is even more difficult since VKC can coexist with sensitization to seasonal pollens. Furthermore, a great variation in clinical score have been showed. So, continue efforts are needed by the scientific community to have a common language on diagnostic criteria, management and treatment. It is also advisable the development of homogeneous scoring system to be routinely used.

### Endocrinology. 1- growth in preterm infants; 2- syndrome of inappropriate secretion of antidiuretic hormone; 3- adrenal hemorrhage; 4- type 1 diabetes

Since early postnatal growth of preterm infants is an important clue in clinical setting, Zhang et al. [13] studied the postnatal growth patterns in a sample of Chinese healthy late preterm infants. Until they reached a term corrected age. Through bivariate, multivariate linear regression analyses and final stepwise regression models, interesting characteristics of postnatal growth have been assessed. Among the most relevant emerge; an extremely low rate (3%) of weight catch down growth, a prevalent weight (46.2%) vs length (30.7%) catch up growth, a faster postnatal weight and length catch up growth in males versus females, as well as in twins versus singletons, a superior weight growth in SGA and AGA versus LGA infants, and a faster length growth velocity in infants of 36 versus 34 and 35 weeks PMA at birth. The results of this study show a better global postnatal growth pattern in late preterm infants than previously described [14–16]. Thus, the authors underline the necessity to consider in follow-up studies the difference in feeding and adopted nutrition strategies, as well as regional, local, ethical and traditional factors that can contribute to the divergence in postnatal growth patterns [16].

The acronym SIADH (syndrome of inappropriate secretion of antidiuretic hormone) indicates a non-physiological secretion of ADH as it occurs independently from effective serum osmolality or circulating blood volume that normally regulates it. ADH stimulates water reabsorption through binding to V2 receptors located in the renal tubules, which mediates the concentration of urine, with relative water excess in plasma leading to hyponatremia. Inappropriate antidiuresis may also result from a gain-of-function mutation in its type 2 receptor. Therefore, some Authors prefer to use the term “syndrome of inappropriate antidiuresis (SIAD)” including both situations. SIADH [17] can be idiopathic or due to multiple causes (neurological, pulmonary, malignant diseases, medications, acute conditions as stress, pain, general anesthesia) because various non-osmotic stimuli may cause AVP release. The classic criteria for diagnosis are those found in the clinical case described by Pintaldi et al. [18] (hyponatremia, high urinary osmolarity, high urinary sodium concentration, absence of edema or clinical signs of volume depletion). However, it is important to remember that the diagnosis of SIADH requires a normal renal, cardiac, hepatic, adrenal and thyroid function [19]. In other words, it is a diagnosis of exclusion, [20] In particular, hypothyroidism (extremely rare) and adrenal insufficiency (AI) must be excluded. AI may be due to ACTH/CRH insufficiency (secondary and tertiary form particularly important in patients who present with neurosurgical conditions, such as traumatic brain injury, subarachnoid hemorrhage and intracranial tumors) or due to a primitive alteration of the adrenal gland which could be congenital or acquired [21, 22]. In fact, in the above mentioned clinical case [18] the analysis of the functional parameters of the ACTH / Cortisol axis led to change the initial diagnosis to the final one of an autoimmune adrenalitis (Addison’s disease) which is considered,after the congenital adrenal hyperplasia (CAH), the second more frequent cause of primary AI. Finally, it is interesting to underline the similarities of the case described by Pintaldi [18] with others reported in Literature [23]. In particular, the initial normality of the values of potassium which would be assumed to be high in case of adrenal insufficiency but which were normal, probably due to the vomiting presented by the patients and in addition, the presence of some slight hyperpigmentation of the skin creases and gingival pigmentations which may be useful for diagnostic orientation during clinical evaluation.

Neonatal adrenal hemorrhage (NAH) is uncommon (0.2–0.55%) [24]. In most cases is asymptomatic and death is rare [25]. Ultrasonographic examination is commonly performed since it is immediate, accurate and safe [26]. In NAH, according to pathological anatomical evolution, temporal modification of echostructure is highly specific. The hemorrhagic adrenal gland is enlarged and homogeneously hyperechoic in newborns. Subsequently, the lysis of the clot increases hypoechoic structure and after 1 or 2 weeks cystic-colliquative feature emerges. Sometimes, shell calcification then appears [27]. An involuted complex structure small mass, partly calcified without vascularization can residue. NAH can regress without relics in a period ranging from 20 days to 5–6 months. Most neonatal supra-renal masses are identified as congenital neuroblastoma (NBL) or adrenal hemorrhage [28]. The histologic examination of tumor tissue or blood marrow besides elevated levels of catecholamine metabolites in urine or serum, is necessary for diagnosis of NBL. Ultrasonographic examination may be useful in assessing adrenal NBL. NBL may appear as a defined small mass within the adrenal gland or an infiltrative complex mass, lobulated with hemorrhagic zones. It is often solid, occasionally with calcific punctate areas in newborns [29]. In NAH, the calcification appears generally later. Ultrasonography differentiation between cystic NAH and cystic NBL may be difficult, especially when catecholamine metabolite values are low. Color Doppler ultrasound examination seems to have most significance in providing a correct diagnosis. In NAH, a nonvascular flow and a regression of lesion over time are observed [30]. In NBL, blood supply is essential for its own growth. This tumor gives rise to characteristic high velocity Doppler shifts. The usual follow-up time for the resolution of the hemorrhage should be within 90 days. NBL should be suspected if the mass does not resolve in 3 months. Adrenal NBL often spontaneously resolve. Therefore, a watchful waiting rather than an interventional approach is suggested [31]. Hypophosphatemia can be one of the consequences of diabetic acidosis (DKA). In most cases of DKA the decrease in blood levels of phosphates is mild, it is not considered responsible for specific pathologies and it does not require correction as various prospective studies have shown no clinical benefit. Phosphate replacement is instead indicated in the severe forms (blood phosphate levels < 1 mg /dL/0.32 mmol / L) with the appearance of symptoms [32]. They can in fact be responsible for clinical manifestations mainly respiratory, neuromuscular, cardiac and hematologic [33]. As regards the cardiac involvement and in particular the arrhythmias, there are no precise epidemiological data but, on the basis of recent patient series, they appear infrequent compared to other complications [34]. In that context, the clinical case described by Miszczuk et al. [35] is an interesting one in particular in two respects: 1) ventricular bigeminy and trigeminy have not been previously described among arrhythmias and although their origin may be multifactorial the fact that they disappear with the phosphate supplementation indicates an important pathogenetic role; 2) the patient studied had blood levels of phosphate slightly higher than the limit considered diagnostic for severe forms, but a set of elements rightly suggest that an intracellular phosphate depletion had been created in the body. In other words, the intracellular phosphate level is primarily responsible for clinical manifestations. Concerning clinical practice, the recent ISPAD consensus concludes: “although administration of phosphate is associated with a risk of hypocalcemia, an IV solution that contains a 50:50 mixture of potassium phosphate and another suitable potassium salt (potassium chloride or potassium acetate), generally permits adequate phosphate replacement while avoiding clinically significant hypocalcemia.”

Mauriac’s syndrome (MS) was initially described in 1930 and it is being characterized by growth failure, delayed puberty, cushingoid appearance, hepatomegaly with abnormal liver enzymes, and hypercholesterolemia. Such a syndrome has been mainly reported in brittle type 1 diabetes (DM1) and very rarely in DM2. In the last few decades, the improvement of diabetes therapy (education, use of insulin and /or its analogues and delivery technology) may have suggested that MS was about the past in the history of diabetes. On the contrary, the most recent reviews in Literature tell us that MS still exists especially in adolescent who do not always accept the rules that a good control of diabetes requires and in patients who live in socially and culturally backward environments where there may be objective difficulties in managing the disease [36]. On the other hand, it should be kept in mind that the hepatomegaly (due to an overloading of hepatocytes with glycogen) represents one of the major signs of MS and it may be the only feature present both in children / adolescents and in adults. It has been defined in various ways: liver glycogen storage, hepatic or liver glycogenosis, DM-associated glycogen storage hepatomegaly and lately “glycogenic hepatopathy” (GH). Real incidence and prevalence of GH are unknown, commonly being misdiagnosed or underdiagnosed [37]. However, if we consider some series of patients, we find that about 10% of diabetic children had hepatic hyperechogeneity decreasing after a period of several months of better adherence to therapy [38]. In other words, hepatomegaly does not seem as rare as generally thought and should be sought in the follow up of diabetic patients. When present, the blood level of transaminases should be assessed, and other causes of liver disease excluded. If there is still a diagnostic doubt, liver biopsy is the gold standard. According to some AA, it would be useful above all to differentiate liver glycogen storage from nonalcoholic fatty liver disease (NAFLD), considering that the former is generally transient and with a benign prognosis while the latter (much more frequent in DM2) can evolve towards forms of cirrhosis and cancer [39, 40].

### Global and public health. 1-violence

Violence against children, adolescent and young adults shows, all over the world, a dramatic and still unsolved concern for the health, the connected social and legal problems. Globally it is estimated that up to1 billion children aged 2–17 years, have experienced physical, sexual, or emotional violence or neglect in one year (as cited in [41] and that in the WHO European Region violence affects over 55 million victims [42]. That can occur in various forms [41]. In Europe we consider prevalences from 9.6% for sexual abuse, 16.3% for physical neglect, 18.4% for emotional neglect, and 2.9% for physical abuse, to 29.6% for emotional abuse [42]. We must also keep in mind that the general prevalence is often underestimated due to the difficulty in various States for fully collecting data. Numerous studies have then highlighted the long-term consequences of the violence suffered. The victims are in fact at greater risk of developing both chronic diseases and behavioural and relational alterations [43]. There are many factors that can facilitate violence both at an individual level (such as lower levels of education, low income, having a disability or mental health problems) and at community level (such as settings with weak governance and poor law enforcement) and with many of such it is possible to act with an appropriate prevention aimed in particular at family and school which are known to be the places where maltreatment occurs most frequently. On the other hand, the importance of prevention has been considered a priority by WHO which in connection with other international agencies has recently proposed the INSPIRE program (www.who.int › inspire-package) based on seven strategies towards reducing and eliminating violence against children. With regard to the school environment in particular, many studies and resulting preventive recommendations have taken into consideration bullying or cyberbullying phenomena which are certainly the most frequent. On the basis of the data collected in Italy by Ferrara et al. [41] and of a recent review of the international literature [44], it is useful to point out that studies about the violent teacher-student relationship are very rare and that this phenomenon, certainly less frequent than the previous one, would deserve greater attention both at the epidemiological level and for establishing setting methods and action to prevent that.

### Neonatology. 1-preterm birth; 2-infant mortality; 3-vitamin K deficiency; 4-Lotus birth

Preterm birth is a public health concern and it represents the leading cause of neonatal mortality [45]. Preterm newborns also present neurological impairment, respiratory, renal and gastrointestinal complications. All these conditions may seriously affect the neurocognitive outcome and cause severe disability. The highest mortality rates of preterm birth have been found in developing countries [46]. In an observational study conducted in Ethiopia, the mortality rate of preterm newborns was 28.8% [47]. Neonatal deaths occurred within the first 24 h in 11.4% of cases and within the first 7 days in 85.23% of instances. Perinatal asphyxia was the major cause of death followed by hyaline membrane disease, jaundice, clinical sepsis and apnoea. Griffin et al. [48] proposed a model for reducing preterm mortality based on WHO recommendations. He pointed out that combined interventions lead to the greatest impact on preterm mortality. The most effective single interventions are oxygen/CPAP, cord care, breastfeeding, and antibiotics. New efforts are needed to prompt identify potential preterm births in order to provide a proper intervention. Granese R et al. [49] found that vaginal/urinary infections, underweight, obesity, unmarried status, uterine anomalies, poly/oligohydramnios, hypertension, diabetes, a history of preterm birth and a short cervix length in the second trimester were the main risk factors for preterm birth. Of note, a short cervix predicted early preterm and very early preterm delivery while the other factors should be considered in late preterm cases. This study indicates that cervix length evaluation [50] during the midtrimester should be performed not only in women at high risk of preterm birth but in all cases since it may early detect high-risk pregnancies and guarantee progesterone administration [51] that diminishes the risk of preterm birth and reduces neonatal morbidity. Socioeconomical factors and health development play a role in the neonatal mortality. In Italy, the overall neonatal mortality rate is 2 per one thousand live births, that is one of the lowest infant mortality rates of all countries. However, there still are differences among areas of the country (North-Centre-South) that should be linked to inadequate care level and organization of perinatal care [52]. Of note, mortality index among immigrants were higher than among Italians. Accordingly, in England ethnic minority newborns had twice risk of adverse events at birth than British infants [53]. Several explanations may be offered to explain these findings. This could be associated with socioeconomical conditions. Language and cultural differences between minority and majority groups can create barriers to access or benefit from information. Newborns, whose parents have a low level of education, are more likely to die in early life [54]. The late onset type of vitamin K deficiency can occur with cerebral haemorrhage. Ceratto and Savino [55] reported a healthy newborn who developed intracranial bleeding due to vitamin K deficiency at the end of the first week of life. She received oral vitamin K at birth because she had no risk factor. These findings can suggest that intramuscular vitamin K may be useful for all newborns because of unpredictable risk factors at birth (malabsorption or cholestasis) other than preterm birth [56]. The American Academy of Pediatrics, the Canadian Pediatric Society [57] and the ESPGHAN Committee on Nutrition [58] recommend the intramuscular administration of the first dose of vitamin K at birth since it would be linked with less failure mainly for late bleeding than oral route administration, due to minor abnormalities of absorption [59]. In many countries, national recommendations on this issue are lacking. There is a need of consistent indications about optimal dose and routes of administration of vitamin K prophylaxis in newborns. Lotus birth is the practice of umbilical nonseverance, leaving the umbilical cord and the placenta intact and attached until it detaches spontaneously after 3–10 days. Meanwhile, the placenta is put in a bowl with salt and herbs [60]. Clinical risks, bioethical and medico legal aspects of this controversial procedure have been analysed by Bonsignore et al. [61]. From the health side, studies about this particular practice are inconsistent, of poor quality and with small sample. There is no clear evidence that having placenta connected to the baby for many days provides benefits to him/her. On the other hand, there is a potential risk for reduced neonatal perfusion and clot formation. In a recent case report series, no infections occurred after lotus birth and mothers expressed interest in repeat lotus birth in the future [62]. However, the dead tissue attached to the newborn may be affected by bacteria and possible complications related to this practice such as omphalitis or other infections have been described [63]. From the medico-legal side, the question arises whether placenta belongs to the mother or to the baby and no clear statement assess the juridical availability of placenta. Therefore, when the mother requests the placenta, she should be allowed to have it, unless a public health issue arises. Families should be adequately updated about the risk/benefit ratio of Lotus birth and informed forms should be used. Overall, Bonsignore et al. [61] consider Lotus birth inadvisable from both medical and rational point of view.

### Nutrition. 1-vitamin D; 2-malnutrition

Vitamin D subclinical deficiency or insufficiency continue to be a common finding [64, 65]. Vitamin D is useful for bone health and it has been investigated for preventing cancer, cardiovascular disease, type 2 diabetes mellitus, neurologic disorders, autoimmune disorders, infectious disease also by increasing the function of gastrointestinal microbiota [66] which may have a role in preventing infectious diseases [67]. Vitamin D supplementation is a matter of debate. It is usually recommended when children have risk factors for deficiency of vitamin D [64] such as inadequate intake, limited skin exposure to sunlight, dark skin, malabsorption, drug intake (anticonvulsants, systemic glucocorticoids, antiretroviral therapy), liver or kidney diseases, obesity). In Italy, the recommended supplementation in children from 1 to 17 years is 600–1000 IU/day vitamin D3 and children with risk factors should receive 1000–1500/UI vitamin D3 per day [64]. To prevent the deficiency of vitamin D, it was conducted a study on the effect of vitamin D3 1500 IU/day [65] from November to April versus no supplementation [64]. In the intervention group, there was no side effect and the 25-hydroxyvitamin D maximum serum level was 71 ng/ml. In adolescents with vitamin D insufficiency, there was only a slight increase in mean 25-hydroxyvitamin D serum level so it is possible that this age range would receive a higher dose of vitamin D3. No consensus has been reached about the period of the administration of vitamin D supplementation [68]. In this study, the intervention group had normal serum value of 25-hydroxyvitamin D all the year except in May. In the control group vitamin D deficiency were found in 4 months out of 6 at the beginning of the year and vitamin D insufficiency in the other 2 months. So, it would be reasonable to extend vitamin D supplementation.

Undernutrition is still a leading cause of morbidity and mortality in children and in some developing countries. Both acute and chronic forms (wasting and stunting) represent a public health challenge. They are strongly inversely correlated with the wealth of nations. In Sub-Saharan Africa [69] and in South Asia [70], risk factors for wasting and stunting in children are low birth weight, low mother’s BMI, small birth size, low parental education, young mother, increasing child’s age, male, inadequate food supply, unhealthy living environments. In the slums of Nairobi (Kenia), 26.30% of children under 12 months of age was stunted, 6.3% wasted and 13.16% underweight [71]. Wasting was more frequently associated with infectious diseases such as cough and rapid breathing and diarrhea, probably due to the acute loss of weight. Regarding mortality, it has been estimated that 45% of deaths in children under 5 years of age in the world is due to undernutrition [72]. Mid-upper arm circumference (MUAC) and weight-for-height/length Z-score (WHZ) are used to identify children with severe acute malnutrition (SAM) but they are affected by gender, region and age bias [73]. Therefore, the burden of SAM can be higher than that reported. Moreover, SAM is a risk factor for growth retardation and impaired psychosocial and cognitive development. Fikrie et al. [74] studied Ethiopian hospitalized children with malnutrition assessed by mid-upper arm circumference (MUAC), weight-for-height/length Z-score (WHZ), edema. All children presented co-morbidities, including pneumonia, diarrhea, anaemia, tuberculosis. They found that the recovery rate was 69.4%, which was below the minimum accepted international standard of 75% [75] and the mortality rate was 10%. Weight gain and length of staying were in line with the international standard and Ethiopian protocol for management of SAM. These results are concordant with those of a similar research conducted in Northwest Ethiopia [76]. Comorbidities in the enrolled population may elucidate the low recovery rate [76]. Other explanations may be related to therapeutic milk intake and high rates of mortality. Finally, relapse after discharge was 7.1% of all cases. This highlights that standardized protocols for SAM follow-up after discharge are always needed.

### Nephrology. 1-congenital anomalies; 2-Hypophosphatemic rickets

Congenital anomalies of the kidney and urinary tract (CAKUTs) include structural and functional abnormalities of the kidney, collecting system, bladder, and urethral abnormalities, and are some of the most common birth defects in newborns. Li and collaborators conducted a retrospective study in China’s Zhejiang Province, including all births and all ascertained patients with CAKUTs registered from 2010 to 2016. There were enrolled 2790 patients identified among 1,748,038 births [77]. Authors observed that males (OR 1.28, 95% CI 1.18–1.38), multiple births (OR 1.53, 95% CI 1.21–1.92) and births in urban areas (OR 1.27, 95% CI 1.18–1.37) presented a higher risk of CAKUTs. Instead, CAKUTs were poorly associated with maternal age. Overall, 22.69% of births with CAKUTs had associated malformations, especially heart defects. The most frequent CAKUT was hydronephrosis, (31.79%), followed by polycystic kidney, renal agenesis, renal ectopia, and renal duplication. In this study, the prevalence of CAKUTs was much lower than in reports from Copenhagen, Russia, and western areas of Saudi Arabia, maybe explained by differences in sociodemographic background, malformation inclusion criteria. In Murmansk County, a population-based birth registry recorded anomalies diagnosed from 22 weeks of gestation to hospital discharge [78]. Those authors admitted that some figures had been overestimated owing to the lack of strict diagnostic criteria for pyelectasis, hydronephrosis, and unspecified anomalies [78]. In Saudi Arabia, the high rate of consanguineous marriage within the local population might increase the rate of CAKUTs [79]. The study in Denmark followed up a birth cohort for 8 years [80]. Finally, the authors evaluated that the prevalence of CAKUTs doubled from 2010 to 2016, which might be owing to increased screening, developments in ultrasound technology, and improved birth defect surveillance. X-linked hypophosphatemic rickets (XLH) is a rare disease caused by mutations in the PHEX gene, this disease is poorly known, and diagnosis is frequently delayed. Emma et al. collected data by means of a questionnaire on XLH epidemiology, diagnosis and treatment, from 10 Italian centres on 175 patients, followed between 1998 and 2017 [81]. The diagnosis was made before the age of 1 and between 1 and 5 years in 11 and 50% of cases, respectively. Clinically apparent bone deformities were present in 95% of patients. Other frequent complications included bone pain (40%), dental abscesses (33%), and dental malpositions (53%). Treatment protocols varied substantially among centres. Nephrocalcinosis, a complication of conventional treatment, was observed in 34% of patients. Tertiary hyperparathyroidism developed in 6% of patients. Overall, nephrocalcinosis has been reported in the literature in 30–70% of patients [82–84]. The present study was conducted to evaluate the current status in the diagnosis and treatment of XLH in Italy. Overall, results are in line with available data in the literature, although with some noticeable differences. The prevalence of XLH is estimated between 1.2–3.0/60,000 [82]. In this survey the number is lower than expected, indicating that the disease is either underdiagnosed.

### Neurology. 1-PANDAS syndrome; 2-headache

Guido et al. showed the successful results of the eye movement desensitisation and reprocessing (EMDR) therapy associated with parent management training (PMT) in a 11-year-old boy who presented with simple and complex vocal tics, motor tics, obsessive-compulsive traits and irritability from the age of 6 years, diagnosed as paediatric autoimmune neuropsychiatric disorder associated with streptococcus (PANDAS) [85]. These results indicate the possibility of improving the treatment outcomes of patients with PANDAS by a combined approach using both antibiotic and EMDR therapies. According to the most recent guidelines [86], elective evidence-based therapies for the treatment of PANDAS include Cognitive Behavioural Therapy, parent training and drug therapy. EMDR therapy had never been utilised before in patients with PANDAS syndrome [87], this method works on the present and not on the past. The explanatory model underlying these considerations is the Adaptive Information Processing model, in which previously stored dysfunctionally without proper assimilation within of a wider adaptive network present in the patient [88].

Migraine is one of the most prevalent chronic pain manifestations of childhood, affecting up to 10% of children between the ages of 5 and 15 years and up to 28% of adolescents aged from 15 to 19 years [89]. Moreover, parents are often concerned about chronic therapies and even clinicians prefer to avoid prescription of prophylactic therapies in children, due to the poor evidence of efficacy and significant potential adverse effects in this population [90]. Moscano et al. conducted an observational multicenter study performed in 91 children with migraine, with (MO) or without aura (MA), or tension-type headache (TTH) [91]. A fixed-dose of Partena® tablets (a combination of Mg2+ 169 mg, CoQ10 20 mg, VitB2 4,8 mg, Feverfew 150 mg-1,2 mg Parthenolides and *Andrographis paniculata* 100 mg), was administered for 16 weeks. The herbal supplement significantly reduced the frequency of headaches in TTH patients during treatment period, maintaining the efficacy after 16 weeks of treatment withdrawal. A significant effect was observed also in the MO and MA groups during treatment. These results are according several epidemiologic, preclinical and clinical evidence supporting the usefulness of other active principles of Partena® in prophylactic treatment of migraine currently available in Europe and USA as dietary supplements [92]. The most recent randomized controlled trial, using a stable extract, add some positive evidence about the efficacy of in the prophylactic treatment of migraine [92]. Studies on Feverfew efficacy in children and adolescents with migraine are lacking.

### Gastroenterology. 1-celiac disease; 2-Alagille syndrome

An increased prevalence of celiac disease (CD) has been observed in several cohorts of cystic fibrosis (CF) patients. A recent study indicates that the gluten/gliadin-derived peptide (P31–43) can cause the cystic fibrosis transmembrane conductance regulator (CFTR) channel protein inhibition in intestinal epithelial cells, thus causing a local stress response that contributes to the immunopathology of CD. Mauri et al. speculated that P31–43-induced CFTR inhibition elicits the danger signals that ignite the epithelial stress response and perturb epithelial proteostasis [93]. Importantly, potentiators of CFTR channel gating, such as the FDA-approved drug Ivacaftor, prevent P31–43 driven CFTR inhibition and suppress the gliadin-induced stress response in cells from celiac patients, as well as the immunopathology developing in gliadin-sensitive mice. Altogether these findings demonstrate that gliadin induced CFTR malfunction is at the apex of the pathogenic cascade leading to CD [94, 95]. Paucity of interlobular bile ducts is an important observation at liver biopsy in the diagnostic work-up of neonatal cholestasis. To date, other than in the Alagille syndrome, syndromic paucity of interlobular bile ducts has been documented in four cholestatic neonates with HFN1β mutations. A syndromic phenotype, known as renal cysts and diabetes syndrome (RCAD), has been identified. Pinon et al. reported a novel case of 5-week-old boy affected by paucity of interlobular bile ducts due to an HFN1β defect [96]. He was admitted for cholestatic jaundice with increased gamma-glutamyl transpeptidase and an unremarkable clinical examination, characterized by cholestatic disease, hyperechogenic kidneys with multiple bilateral cortical cysts at ultrasound examination, associated with moderately impaired renal function with proteinuria, polyuria and metabolic acidosis, paucity of interlobular bile ducts at liver biopsy, thus the diagnosis of Alagille syndrome (AGS) was considered, but excluded. Although genetic tests for liver cholestatic diseases were performed with negative results for Alagille syndrome (JAG1 and NOTCH2), a de-novo missense mutation of HNF1β gene was detected. To date, only others 5 cases of neonatal cholestasis are reported in literature as associated to HNF1β mutations, in most cases de-novo deletions, with similar clinical course [97–100]. HFN1β defects should be considered in neonates with cholestasis and renal impairment, especially in SGA and IUGR newborns with a family history of renal disease or diabetes, in addition to AGS.

### Respiratory diseases. 1- recurrent wheezing; 2- Bronchopulmonary dysplasia; 3- cystic fibrosis

Recurrent wheezing and/or asthma are common chronic respiratory disease in children. Studies have demonstrated that children hospitalized for RSV bronchiolitis during infancy were more likely to have subsequent episodes of wheezing [101]. Also, eosinophil-derived neurotoxin (EDN), contained in eosinophil cytotoxic granule proteins has been considered to be involved in the recurrent wheezing and asthma development in later life [102]. Zhai et al. followed-up for 1-year 145 children of 3 years old or younger, who were hospitalized with wheezing, in order to analyse factors that may predict recurrent wheezing [103]. The authors demonstrated that eczema, respiratory syncytial virus (RSV) infection, eosinophil count and eosinophil-derived neurotoxin (EDN) concentration were all risk factors related to recurrent wheezing, speculating that the combination of eosinophil count and serum EDN quantification may be served as one of the biomarkers to predict the recurrent wheezing in clinical practice. This data are confirmed by a double-blind randomized, placebo-controlled study, where the parallel comparison of montelukast and placebo administered for 3 months in 200 infants (age, 6–24 months), who were hospitalized with their first episode of acute RSV bronchiolitis, showed that serum EDN levels correlated significantly with the total number of wheezing episodes at 12 months in both groups of treated with placebo or leukotriene receptor antagonist [104]. There are not standard criteria for weaning from continuous positive airways pressure (CPAP) and/or oxygen therapy the premature babies. Vento et al. wanted to verify if a physiologic test, modified respect to that developed by Walsh and collaborators for estimating bronchopulmonary dysplasia (BPD) rate, can be used as a clinical tool for weaning the premature babies from CPAP and/or oxygen therapy [105, 106]. They tested 23 neonates with body weight (BW) 500–1250 g and gestational age (GA) ≤ 32 weeks, receiving FiO2 ≤ 0.30 by hood or CPAP, monitoring transcutaneous partial pressure of CO2 (TcPCO2) and SpO2, at 28 days of life and at 36 weeks of postmenstrual age, in 3 steps: baseline, challenge (FiO2 and CPAP reduction to room air) and post-test (room air). Six of 23 tested babies (26%) passed the challenge at 28 days of life, 4 of 10 tested babies (40%) passed the challenge at 36 weeks. Median values of SpO2 were significantly higher in the neonates passing the test, respect to the failing patients. At the same time median values of TcPCO2 were significantly higher in the latter babies. The authors speculated that TcPCO2 monitoring appeared to be a new useful parameter for failure prediction of weaning. These data are confirmed in a multicentre study conducted by Kaempf et al., where pCO2 and SpO2 values appears to be reasonably good markers of lung injury, median pCO2 values were significantly higher in infants with BPD compared to controls [107]. Vitamin D plays an important role in inflammatory responses after antigen exposure. T helper 17 cells produce Il-17A promoted by Il-23 and bind it to its receptor on the T cell membrane. They are both critical for neutrophil recruitment in a chronic *P. aeruginosa* pulmonary infection [108]. Olszowiec-Chlebna et al. [109]. conducted a randomized, placebo-controlled, double-blind, cross-over trial in 23 patients with cystic fibrosis (CF), chronically infected by *P. aeruginosa*, and randomly assigned to calcitriol or cholecalciferol groups. The results showed that both analogs of vitamin D revealed their anti-inflammatory effect, reducing the level of Il-17A and Il-23 in the airway of CF patients with chronic *P. aeruginosa* infection, and that calcitriol improve calcium phosphorus metabolism after supplementation without adverse effects. Pincikova et al. randomized CF patients to receive 35,000–50,000 IU vitamin D per week for 3 months and observed that this supplementation has pleiotropic immunomodulatory effects in CF in a dose-dependent manner, demonstrated that free serum 25 OH D level correlated positively with anti-inflammatory soluble immunological parameters [110]. Instead, Olszowiec-Chlebna et al. did not observe any statistically significant changes of 25OHD serum level due to the supplementation with cholecalciferol 1000 IU per day. This is probably related to low dose of administered cholecalciferol.

### Rheumatic diseases

Thrombotic thrombocytopenic purpura (TTP) is a disorder of the blood-coagulation system. Although TTP in patients with systemic lupus erythematosis (SLE) is rare, TTP-SLE has high mortality, ranging from 34 to 62.5% [111]. TTP-SLE is related to endothelial injury or platelet aggregation that lead to vascular injury or autoimmune response. Li et al. want to report the clinical features of patients with TTP-SLE and enrolled 25 paediatric patients (median age 14 years old) [112]. They observed that all patients had decreased platelet count and microangiopathic haemolytic anemia. Fever, rash, edema and neurological symptoms were the main clinical symptoms. Nineteen patients (76%) had impaired renal function, with a lupus nephritis class IV (20%) and thrombotic microangiopathy (20%) at renal biopsy, in line with the observations in adult TTP-SLE patients [113]. Thirteen patients (52%) were treated with glucocorticoids in combination with immunosuppressive agent, and 10 patients (40%) were treated with plasma exchange combined with glucocorticoids plus immunosuppressive agent. One patient died due to lung infection; others had disease remission. These data showed that TTP-SLE often had a moderate to severe lupus disease activity, as confirmed in literature [114]. Testing of LDH level and blood smear should be performed when kidney and neurological symptoms arise in children with SLE. The use of combination therapy, glucocorticoids plus immunosuppressive agent, provided satisfactory clinical outcome. Patients with refractory TTP-SLE will also need plasma exchange therapy.

## Conclusions

The first semester of year 2019 was a remarkable time in the field of paediatrics. We have described developments that have improved our knowledge across many areas. They covered mechanisms and clinical management of diseases. We look forward to additional thought-provoking facts in the future.

## Data Availability

Data sharing is not applicable to this article as no datasets were generated or analyzed during the current study.

## References

[1] Caffarelli C, Dondi A, Povesi Dascola C, Ricci G (2013). Skin prick test to foods in childhood atopic eczema: pros and cons. Ital J Pediatr.

[2] Crisafulli G, Caminiti L, Chiera F, Arasi S, Salzano G, Panasiti I (2019). Omalizumab in children with severe allergic disease: a case series. Ital J Pediatr.

[3] Mastrorilli C, Posa D, Cipriani F, Caffarelli C (2016). Asthma and allergic rhinitis in childhood: what's new. Pediatr Allergy Immunol.

[4] Mastrorilli C, Caffarelli C, Hoffmann-Sommergruber K (2017). Food allergy and atopic dermatitis: prediction, progression, and prevention. Pediatr Allergy Immunol.

[5] Zinelli C, Caffarelli C, Strid J, Jaffe A, Atherton DJ (2009). Measurement of nitric oxide and 8-isoprostane in exhaled breath of children with atopic eczema. Clin Exp Dermatol.

[6] Di Rienzo V, Cadario G, Grieco T, Galluccio AG, Caffarelli C, Liotta G (2014). Sublingual immunotherapy in mite-sensitized children with atopic dermatitis: a randomized, open, parallel-group study. Ann Allergy Asthma Immunol.

[7] Caffarelli C, Garrubba M, Greco C, Mastrorilli C, Povesi Dascola C (2016). Asthma and food allergy in children: is there a connection or interaction?. Front Pediatr.

[8] Carpenter DM, Stover A, Slota C, Ayala GX, Yeatts K, Tudor G (2014). An evaluation of physicians’ engagement of children with asthma in treatment-related discussions. J Child Health Care.

[9] Cappuccio A, Bugliaro F, Caimmi SME, Caldarelli V, Caminiti L, D’Auria E (2019). Consensus communication strategies to improve doctor-patient relationship in paediatric severe asthma. Ital J Pediatr.

[10] Vichyanond P, Pacharn P, Pleyer U, Leonardi A (2014). Vernal keratoconjunctivitis: a severe allergic eye disease with remodeling changes. Pediatr Allergy Immunol.

[11] Zicari AM, Capata G, Nebbioso M, De Castro G, Midulla F, Leonardi L (2019). Vernal Keratoconjunctivitis: an update focused on clinical grading system. Ital J Pediatr.

[12] Cipriani F, Mastrorilli C, Tripodi S, Ricci G, Perna S, Panetta V (2018). Diagnostic relevance of IgE sensitization profiles to eight recombinant Phleum pratense molecules. Allergy..

[13] Zhang L, Li Y, Liang S, Liu X-J, Kang F-L, Li G-M (2019). Postnatal length and weight growth velocities according to Fenton reference and their associated perinatal factors in healthy late preterm infants during birth to term-corrected age: an observational study. Ital J Pediatr.

[14] Fenton TR, Nasser R, Eliasziw M, Kim JH, Bilan D, Sauve R (2013). Validating the weight gain of preterm infants between the reference growth curve of the fetus and the term infant. BMC Pediatr.

[15] University of Calgary. https://ucalgary.ca/resource/preterm-growth-chart/2013chart?utm_source=fenton&utm_medium=redirect&utm_campaign=redirect. Accessed 20 May 2020.

[16] de Onis M, Garza C, Victora CG, Onyango AW, Frongillo EA, Martines J (2004). The WHO multicentre growth reference study: planning, study design, and methodology. Food Nutr Bull.

[17] Cuesta M, Thompson CJ (2016). The syndrome of inappropriate antidiuresis (SIAD). Best Pract Res Clin Endocrinol Metab.

[18] Pintaldi S, Lora A, Vecchiato K, Taddio A, Barbi E (2019). SIADH versus adrenal insufficiency: a life-threatening misdiagnosis. Ital J Pediatr.

[19] Jones DP (2018). Syndrome of inappropriate secretion of antidiuretic hormone and Hyponatremia. Pediatr Rev.

[20] Fenske W, Allolio B (2010). The syndrome of inappropriate secretion of antidiuretic hormone: diagnostic and therapeutic advances. Horm Metab Res.

[21] Bowden SA, Henry R (2018). Pediatric adrenal insufficiency: diagnosis, management, and new therapies. Int J Pediatr.

[22] Buonocore F, Achermann JC (2020). Primary adrenal insufficiency: New genetic causes and their long-term consequences. Clin Endocrinol.

[23] Thompson MD, Kalmar E, Bowden SA (2015). Severe hyponatraemia with absence of hyperkalaemia in rapidly progressive Addison’s disease. BMJ Case Rep.

[24] Lee MC, Lin LH (2000). Utrasound screening of neonatal adrenal hemorrhage. Acta Pediatr Taiwan.

[25] Miele V, Galluzzo M, Pedicelli C, Adami L, Calisti A (2000). A neonatal adrenal hemorrhage associated with scrotal hematoma. Radiol Med.

[26] Kawashima A, Sandler CM, Ernst RD, Takahashi N, Roubidoux MA, Goldman SM (1999). Imaging of non traumatic hemorrhage of adrenal gland. Radiographics..

[27] Wang CH, Chen AJ, Yang LY, Tang RB (2008). Neonatal adrenal hemorrhage presenting as a multiloculate cystic mass. J Chin Med Assoc.

[28] Postek G, Streich H, Narebeski K (2011). Assessment of diagnostic methodsin adrenal gland hemmorage in neonates on the basis of own material from the years 2007-2011. Pol J Radiol.

[29] Eklof O, Mortensson W, Sandsteadt B (1986). Suprarenal haematoma versus neuroblastoma complicated by haemorrhages. A diagnostic dilemma in the newborn. Acta Radiol Diagn.

[30] Patankar JZ, Mali VP, Prabhakaran K (2002). Neonatal adrenal hemorrhagic pseudocyst. J Postgrad Med.

[31] Eo H, Kim JH, Jang KM, Yoo SY, Lim GY, Kim MJ (2011). Comparision of clinical-radiological features between congenital cystic neuroblastoma and neonatal adrenal hemorrhagic pseudocyst. Korean J Radiol.

[32] Wolfsdorf JI, Glaser N, Agus M, Fritsch M, Hanas R, Rewers A (2018). ISPAD clinical practice consensus guidelines 2018: diabetic ketoacidosis and the hyperglycemic hyperosmolar state. Pediatr Diabetes.

[33] Ditzel J, Lervang HH (2010). Disturbance of inorganic phosphate metabolism in diabetes mellitus: clinical manifestations of phosphorus-depletion syndrome during recovery from diabetic ketoacidosis. Diab Metab Syndr Obes.

[34] Abbas Q, Arbab S, Haque AU, Humayun KN (2018). Spectrum of complications of severe DKA in children in pediatric intensive care unit. Pak J Med Sci.

[35] Miszczuk K, Mroczek-Wacinska J, Piekarski R, Wysocka-Lukasik B, Jawniak R, Ben-Skowronek I (2019). Ventricular bigeminy and trigeminy caused by hypophosphataemia during diabetic ketoacidosis treatment: a case report. Ital J Pediatr.

[36] Lombardo F, Passanisi S, Gasbarro A, Tuccari G, Ieni A, Salzano G (2019). Hepatomegaly and type 1 diabetes: a clinical case of Mauriac's syndrome. Ital J Pediatr.

[37] Aluko A, Enofe I, Burch J, Yam J, Khan N (2020). Hepatocellular glycogen accumulation in the setting of poorly controlled type 1 diabetes mellitus: case report and review of the literature. Case Rep Hepatol.

[38] Al-Hussaini AA, Sulaiman NM, Alzahrani MD, Alenizi AS, Khan M (2012). Prevalence of hepatopathy in type 1 diabetic children. BMC Pediatr.

[39] Lui DT, Woo YC, Chow WS, Lee C-H, Lee ACH, Leung EKH (2019). Glycogenic hepatopathy as an unusual etiology of deranged liver function in a patient with type 1 diabetes: a case report. Medicine (Baltimore).

[40] Patita M, Nunes G, Alves de Matos A, Coelho H, Fonseca C, Fonseca J (2019). Mauriac syndrome: a rare hepatic glycogenosis in poorly controlled type 1 diabetes. GE Port J Gastroenterol.

[41] Ferrara P, Franceschini G, Villani A, Corsello G (2019). Physical, psychological and social impact of school violence on children. Ital J Pediatr.

[42] Sethi D, Bellis M, Hughes K, Gilbert R, Mitis F, Galea G (2013). European report on preventing child maltreatment.

[43] Hughes K, Bellis MA, Hardcastle KA, Sethi D, Butchart A, Mikton C (2017). The effect of multiple adverse childhood experiences on health: a systematic review and meta-analysis. Lancet Public Health.

[44] Lester S, Lawrence C, Warda CL (2017). What do we know about preventing school violence? A systematic review of systematic reviews. Psychol Health Med.

[45] Suff N, Story L, Shennan A (2019). The prediction of preterm delivery: what is new?. Semin Fetal Neonatal Med.

[46] Chawanpaiboon S, Vogel JP, Moller AB, Lumbiganon P, Petzold M, Hogan D (2019). Global, regional, and national estimates of levels of preterm birth in 2014: a systematic review and modelling analysis. Lancet Glob Health.

[47] Yismaw AE, Gelagay AA, Sisay MM (2019). Survival and predictors among preterm neonates admitted at University of Gondar comprehensive specialized hospital neonatal intensive care unit, Northwest Ethiopia. Ital J Pediatr.

[48] Griffin JB, Jobe AH, Rouse D, McClure EM, Goldenberg RL, Kamath-Rayne BD (2019). Evaluating WHO-recommended interventions for preterm birth: a mathematical model of the potential reduction of preterm mortality in sub-Saharan Africa. Glob Health Sci Pract.

[49] Granese R, Gitto E, D’Angelo G, Falsaperla R, Corsello G, Amadore D (2019). Preterm birth: seven-year retrospective study in a single centre population. Ital J Pediatr.

[50] Boeling RC, Dugoff L, Roman A, Berghella V, Ludmir J (2019). Predicting asymptomatic cervical dilation in pregnant patients with short mid-trimester cervical length: a secondary analysis of a randomized controlled trial. Acta Obstet Gynecol Scand.

[51] Jarde A, Lutsiv O, Beyene J, McDonald SD (2019). Vaginal progesterone, oral progesterone, 17-OHPC, cerclage, and pessary for preventing preterm birth in at-risk singleton pregnancies: an updated systematic review and network meta-analysis. BJOG..

[52] Simeoni S, Frova L, De Curtis M (2019). Inequalities in infant mortality in Italy. Ital J Pediatr.

[53] Opondo C, Gray R, Hollowell J, Li Y, Kurinczuk JJ, Quigley MA (2019). Joint contribution of socioeconomic circumstances and ethnic group to variations in preterm birth, neonatal mortality and infant mortality in England and Wales: a population-based retrospective cohort study using routine data from 2006 to 2012. BMJ Open.

[54] Braudt DB, Lawrence E, Tilstra A, Rogers R, Hummer R (2019). Family socioeconomic status and early life mortality risk in the United States. Matern Child Health J.

[55] Ceratto S, Savino F (2019). Vitamin K deficiency bleeding in an apparently healthy newborn infant: the compelling need for evidence-based recommendation. Ital J Pediatr.

[56] Witt M, Kvist N, Jørgensen MH, Hulscher JB, Verkade HJ (2016). Netherlands study group of biliary atresia registry (NeSBAR). Prophylactic dosing of vitamin K to prevent bleeding. Pediatrics.

[57] Ng E, Loewy AD (2018). Guidelines for vitamin K prophylaxis in newborns. Paediatr Child Health.

[58] Mihatsch WA, Braegger C, Bronsky J, Campoy C, Domellöf M, Fewtrell M (2016). Prevention of vitamin K deficiency bleeding in newborn infants: a position paper by the ESPGHAN committee on nutrition. J Pediatr Gastroenterol Nutr.

[59] Araki S, Shirahata A (2020). Vitamin K deficiency bleeding in infancy. Nutrients..

[60] Zinsser LA (2018). Lotus birth, a holistic approach on physiological cord clamping. Women Birth.

[61] Bonsignore A, Buffelli F, Ciliberti R, Ventura F, Molinelli A, Fulcheri E (2019). Medico-legal considerations on “Lotus birth” in the Italian legislative framework. Ital J Pediatr.

[62] Monroe KK, Rubin A, Mychaliska KP, Skoczylas M, Burrows HL (2019). Lotus birth: a case series report on umbilical nonseverance. Clin Pediatr (Phila).

[63] Steer-Massaro C (2020). Neonatal omphalitis after Lotus birth. J Midwifery Womens Health.

[64] Mazzoleni S, Magni G, Toderini D (2019). Effect of vitamin D3 seasonal supplementation with 1500 IU/day in north Italian children (DINOS study). Ital J Pediatr.

[65] Holick MF (2010). The D-lemma: to screen or not to screen for 25-hydroxyvitamin D concentrations. Clin Chem.

[66] Jin D, Wu S, Zhang YG, Lu R, Xia Y, Donget H (2015). Lack of vitamin D receptor causes dysbiosis and changes the functions of the murine intestinal microbiome. Clin Ther.

[67] Caffarelli C, Cardinale F, Povesi-Dascola C, Dodi I, Mastrorilli V, Ricci G (2015). Use of probiotics in pediatric infectious diseases. Expert Rev Anti-Infect Ther.

[68] Spector TD, Lewis L (2016). Should healthy people take a vitamin D supplement in winter months?. BMJ.

[69] Akombi BJ, Agho KE, Hall JJ, Wali N, Renzaho AMN, Merom D (2017). Stunting, wasting and underweight in sub-Saharan Africa: a systematic review. Int J Environ Res Public Health.

[70] Akhtar S (2016). Malnutrition in South Asia—a critical reappraisal. Crit Rev Food Sci Nutr.

[71] De Vita M, Scolfaro C, Santini B, Lezo A, Gobbi F, Buonfrate D (2019). Malnutrition, morbidity and infection in the informal settlements of Nairobi, Kenya: an epidemiological study. Ital J Pediatr.

[72] Adebisi YA, Ibrahim K, Lucero-Prisno DE, Ekpenyong A, Micheal AI, Chinemelumet IG (2019). Prevalence and socio-economic impacts of malnutrition among children in Uganda. Nutr Metab Insights.

[73] Tessema M, Laillou A, Tefera A, Teklu Y, Berger J, Wieringa FT (2020). Routinely MUAC screening for severe acute malnutrition should consider the gender and age group bias in the Ethiopian nonemergency context. PLoS One.

[74] Fikrie A, Alemayehu A, Gebremedhin S (2019). Treatment outcomes and factors affecting time-to-recovery from severe acute malnutrition in 6–59 months old children admitted to a stabilization center in southern Ethiopia: a retrospective cohort study. Ital J Pediatr.

[75] Sphere-Project (2011). The sphere project, Humanitarian charter and minimum standards in humanitarian response.

[76] Wondim A, Tigabu B, Kelkay MM (2020). Time to recovery from severe acute malnutrition and its predictors among admitted children aged 6-59 months at the therapeutic feeding center of Pawi General Hospital, Northwest Ethiopia: a retrospective follow-up study. Int J Pediatr.

[77] Li Z, Chen Y, Qiu L, Chen D, Hu C, Xu J (2019). Prevalence, types, and malformations in congenital anomalies of the kidney and urinary tract in newborns: a retrospective hospital-based study. Ital J Pediatr.

[78] Postoev VA, Grjibovski AM, Kovalenko AA, Anda EE, Nieboer E, Odland JO (2016). Congenital anomalies of the kidney and the urinary tract: a Murmansk County birth registry study. Birth Defects Res A Clin Mol Teratol.

[79] Bondagji NS (2014). Antenatal diagnosis, prevalence and outcome of congenital anomalies of the kidney and urinary tract in Saudi Arabia. Urol Ann.

[80] Andrés-Jensen L, Jørgensen FS, Thorup J, Flachs J, Madsen JL, Marounet LL (2016). The outcome of antenatal ultrasound diagnosed anomalies of the kidney and urinary tract in a large Danish birth cohort. Arch Dis Child.

[81] Emma F, Cappa M, Antoniazzi F, Bianchi ML, Chiodini I, Eller Vainicher C (2019). X-linked hypophosphatemic rickets: an Italian experts’ opinion survey. Ital J Pediatr.

[82] Rafaelsen S, Johansson S, Raeder H (2016). Hereditary hypophosphatemia in Norway: a retrospective population-based study of genotypes, phenotypes, and treatment complications. Eur J Endocrinol.

[83] Makitie O, Doria A, Kooh SW, Cole WG, Daneman A, Sochett E (2003). Early treatment improves growth and biochemical and radiographic outcome in X-linked hypophosphatemic rickets. J Clin Endocrinol Metab.

[84] Verge CF, Lam A, Simpson JM, Cowell CT, Howard NJ, Silink M (1991). Effects of therapy in X-linked hypophosphatemic rickets. N Engl J Med.

[85] Guido CA, Zicari AM, Duse M, Spalice A (2019). Eye movement desensitisation and reprocessing (EMDR) treatment associated with parent management training (PMT) for the acute symptoms in a patient with PANDAS syndrome: a case report. Ital J Pediatr.

[86] Swedo SE, Frankovich J, Murphy TK (2017). Overview of treatment of pediatric acute-onset neuropsychiatric syndrome. J Child Adolesc Psychopharmacol.

[87] Moreno-Alcázar A, Treen D, Valiente-Gómez A, Sio-Eroles A, Pérez V, Amann BL (2017). Efficacy of eye movement desensitization and reprocessing in children and adolescent with post-traumatic stress disorder: a meta-analysis of randomized controlled trials. Front Psychol.

[88] Landin-Romero R, Novo P, Vicens V, McKenna PJ, Santed A, Pomarol-Clotetet E (2013). EMDR therapy modulates the default mode network in a subsyndromal, traumatized bipolar patient. Neuropsychobiology.

[89] Kacperski J, Bazarsky A (2017). New developments in the prophylactic drug treatment of pediatric migraine: what is new in 2017 and where does it leave us?. Curr Pain Headache Rep.

[90] Powers SW, Coffey CS, Chamberlin LA, Ecklund DJ, Klingner EA, Yankeyet JW (2017). Trial of amitriptyline, topiramate, and placebo for pediatric migraine. N Engl J Med.

[91] Moscano F, Guiducci M, Maltoni L, Striano P, Ledda MG, Zoroddu F (2019). An observational study of fixed-dose Tanacetum parthenium nutraceutical preparation for prophylaxis of pediatric headache. Ital J Pediatr.

[92] Daniel O, Mauskop A (2016). Nutraceuticals in acute and prophylactic treatment of migraine. Curr Treat Options Neurol.

[93] Maiuri L, Villella VR, Raia V, Kroemer G (2019). The gliadin-CFTR connection: new perspectives for the treatment of celiac disease. Ital J Pediatr.

[94] Villella VR, Venerando A, Cozza G, Esposito S, Ferrari E, Monzaniet R (2019). A pathogenic role for cystic fibrosis transmembrane conductance regulator in celiac disease. EMBO J.

[95] Maiuri L, Villella V, Piacentini M, Raia V, Kroemer G (2019). Defective proteostasis in celiac disease as a new therapeutic target. Cell Death Dis.

[96] Pinon M, Carboni M, Colavito D, Cisarò F, Peruzzi L, Pizzol A (2019). Not only Alagille syndrome. Syndromic paucity of interlobular bile ducts secondary to HNF1β deficiency: a case report and literature review. Ital J Pediatr.

[97] Raile K, Klopocki E, Holder M, Wessel T, Galler A, Deiss D (2009). Expanded clinical spectrum in hepatocyte nuclear factor 1b-maturity-onset diabetes of the young. J Clin Endocrinol Metab.

[98] Kotalova R, Dusatkova P, Cinek O, Dusatkova L, Dedic T, Seeman T (2015). Hepatic phenotypes of HNF1B gene mutations: a case of neonatal cholestasis requiring portoenterostomy and literature review. World J Gastroenterol.

[99] Beckers D, Bellanné-Chantelot C, Maes M (2007). Neonatal cholestatic jaundice as the first symptom of a mutation in the hepatocyte nuclear factor-1beta gene (HNF-1beta). J Pediatr.

[100] Kitanaka S, Miki Y, Hayashi Y, Igarashi T (2004). Promoter-specific repression of hepatocyte nuclear factor (HNF)-1 beta and HNF-1 alpha transcriptional activity by an HNF-1 beta missense mutant associated with type 5 maturityonset diabetes of the young with hepatic and biliary manifestations. J Clin Endocrinol Metab.

[101] Henderson J, Hilliard TN, Sherriff A, Stalker D, Al Shammari N, Thomas HM (2005). Hospitalization for RSV bronchiolitis before 12 months of age and subsequent asthma, atopy and wheeze: a longitudinal birth cohort study. Pediatr Allergy Immunol.

[102] McBrien CN, Menzies-Gow A (2017). The biology of eosinophils and their role in asthma. Front Med.

[103] Zhai J, Zou Y, Liu J, Jin X, Ma C, Li J (2019). Analysis of the predicting factors of recurrent wheezing in infants. Ital J Pediatr.

[104] Kim CK, Choi J, Kim HB, Callaway Z, Shin BM, Kim JT (2010). A randomized intervention of montelukast for post-bronchiolitis: effect on eosinophil degranulation. J Pediatr.

[105] Vento G, Vendettuoli V, Aurilia C, Tana M, Tirone C, Lio A (2019). A modified physiologic test for bronchopulmonary dysplasia: a clinical tool for weaning from CPAP and/or oxygen-therapy the premature babies?. Ital J Pediatr.

[106] Walsh MC, Wilson-Costello D, Zadell A, Newman N, Fanaroff A (2003). Safety, reliability, and validity of a physiologic definition of bronchopulmonary dysplasia. J Perinatol.

[107] Kinsella JP, Greenough A, Abman SH (2006). Bronchopulmonary dysplasia. Lancet..

[108] Dubin PJ, Martz A, Eisenstatt JR, Fox MD, Logar A, Kolls JK (2012). Interleukin-23- mediated inflammation in *Pseudomonas aeruginosa* pulmonary infection. Infect Immun.

[109] Olszowiec-Chlebna M, Koniarek-Maniecka A, Brzozowska A, Błauż A, Rychlik B, Stelmach I (2019). Vitamin D inhibits pro-inflammatory cytokines in the airways of cystic fibrosis patients infected by Pseudomonas aeruginosa- pilot study. Ital J Pediatr.

[110] Pincikova T, Paquin-Proulx D, Sandberg JK, Flodström-Tullberg M, Hjelte L (2017). Vitamin D treatment modulates immune activation in cystic fibrosis. Clin Exp Immunol.

[111] Letchumanan P, Ng HJ, Lee LH, Thumboo J (2009). A comparison of thrombotic thrombocytopenic purpura in an inception cohort of patients with and without systemic lupus erythematosus. Rheumatology (Oxford).

[112] Li J, Jiang J, Wang C, Jian S, Zhou Y, Ma MS (2019). Clinical features and prognosis of patients with thrombotic thrombocytopenic purpura associated with systemic lupus erythematosus: a review of 25 cases. Ital J Pediatr.

[113] Jiang H, An X, Li Y, Sun Y, Shen G, Tu Y (2014). Clinical features and prognostic factors of thrombotic thrombocytopenic purpura associated with systemic lupus erythematosus: a literature review of 105 cases from 1999 to 2011. Clin Rheumatol.

[114] Joly BS, Coppo P, Veyradier A (2018). Pediatric thrombotic thrombocytopenic purpura. Eur J Haematol.

